# Orbital apex syndrome affecting head and neck cancer patients: A case series

**DOI:** 10.4317/medoral.21506

**Published:** 2017-04-08

**Authors:** Ana-Carolina Prado-Ribeiro, Ana-Claudia Luiz, Marco-Aurelio Montezuma, Milena-Perez Mak, Alan- Roger Santos-Silva, Thaís-Bianca Brandão

**Affiliations:** 1 Dental Oncology Service, Instituto do Câncer do Estado de São Paulo, ICESP, Faculdade de Medicina da Universidade de São Paulo, São Paulo, Brazil; 2 Clinical Oncology Service, Instituto do Câncer do Estado de São Paulo, ICESP, Faculdade de Medicina da Universidade de São Paulo, São Paulo, Brazil; 3 Oral Diagnosis Department, Semiology Area, Piracicaba Dental School, University of Campinas (UNICAMP), Piracicaba, Sao Paulo, Brazil

## Abstract

**Background:**

Orbital apex syndrome (OAS) is a complex and uncommon disorder that typically damages multiple cranial nerves in association with optic nerve dysfunction. OAS is associated with several different pathologies, however; only a few cases have been reported in association with head and neck cancer (HNC) so far.

**Material and Methods:**

A case series of HNC patients diagnosed with OAS is described including clinicopathological data, image findings, and disease outcome.

**Results:**

Ptosis and diplopia were diagnosed in four male patients with mean age of 61.2 years who were undergoing treatment for late-stage carcinomas of the tongue, larynx, and nasopharynx, eventually leading to the diagnosis of OAS. The mean overall survival rate after the diagnosis of OAS was 9.5 months.

**Conclusions:**

The current study reinforces evidence that OAS indicates poor prognosis and highlights the importance of early diagnosis.

** Key words:**Head and neck cancer, oral cancer, metastasis, orbital apex syndrome, optic neuropathy.

## Introduction

Orbital apex syndrome (OAS) is a rare disorder characterized by the involvement and damage of cranial nerves, including the oculomotor (III), trochlear (IV), abducens (VI) and ophthalmic branch of the trigeminal nerve (V) in association with optic dys-function (1-3). OAS may be associated with inflammatory diseases (sarcoidosis, systemic lupus, Wegener granulomatosis), fungal infections (*Mucormycosis*, *Aspergillus*), bacterial infections (*Actinomyces*, *Mycobacterium tuberculosis*), viral infections (*Herpes zoster*), primary and metastatic tumors (nasopharyngeal carcinoma, neural tumors, metastatic tumors, lymphomas and leukemia) as well as trauma (sinonasal surgery), among others (mucoceles) (1,2).

From a clinical point of view, patients diagnosed with OAS initially present visual loss and ophthalmoplegia, such as proptosis, ptosis, fixed dilated pupil, and diplopia. Associated periorbital pain may also develop in these patients (1-3).

Apparently, the association of OAS and head and neck cancer (HNC) is considered to be exceptionally rare, with only a few cases published in the English-related literature so far (4,5). Therefore, the aim of the present case series was to report four additional cases of OAS diagnosed in patients undergoing treatment for advanced carcinomas of the head and neck region, including tumors of the tongue, larynx, and nasopharyns. In addition, clinic and image data relevant for diagnosis will be discussed in the light of the scientific literature pertinent to OAS.

## Material and Methods

This is a retrospective case description report based on patients who were undergoing dental treatment at the Dental Oncology Service of the Instituto do Cancer do Estado de Sao Paulo (ICESP), from March 2011 to January 2013. In order to be included in this case series, the patients had to present a confirmed diagnosis of HNC and develop ocular alterations or ophthalmoplegia. Complete medical information including demographic and clinicopathologic data, diagnostic head and neck imaging and post-cancer treatment follow-up also had to be complete and fully available at the patients’ medical charts.

This retrospective study was reviewed and approved by the Ethics Committee of the University of Sao Paulo Medical School, Sao Paulo, Brazil (study protocol number 882,731).

## Results

**Figure 1:**
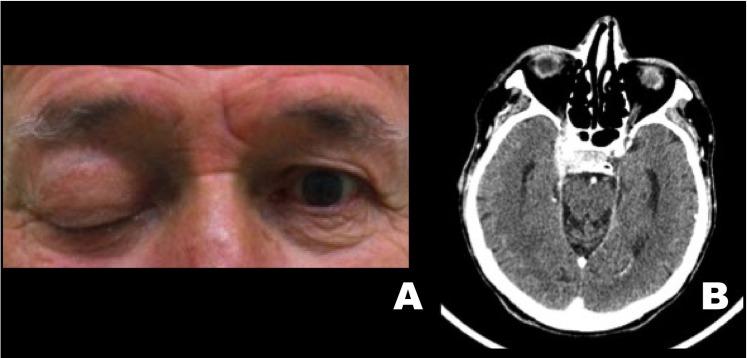
A. Extraoral clinical aspect showing right eyelid ptosis. B. Axial CT (soft-tissue window display) demonstrating an extensive hyperdense image at the base of the skull associated with the sphenoid bone.

**Figure 2:**
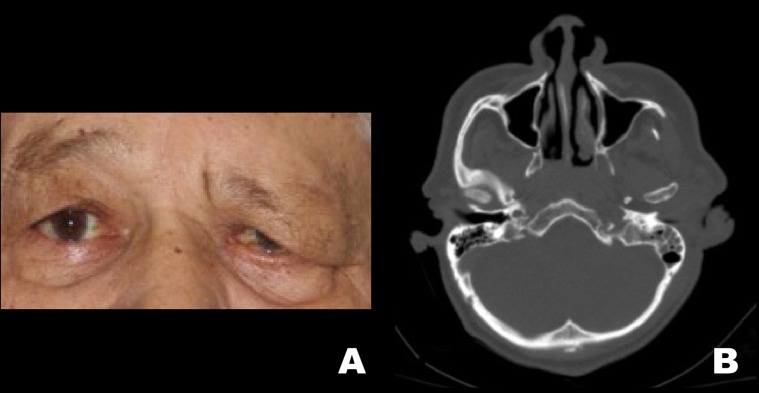
A. Extraoral clinical image of the left eyelid ptosis. B. Axial CT (bone-window display) showing a thickening of the left pinna and external ear conduit.

**Figure 3:**
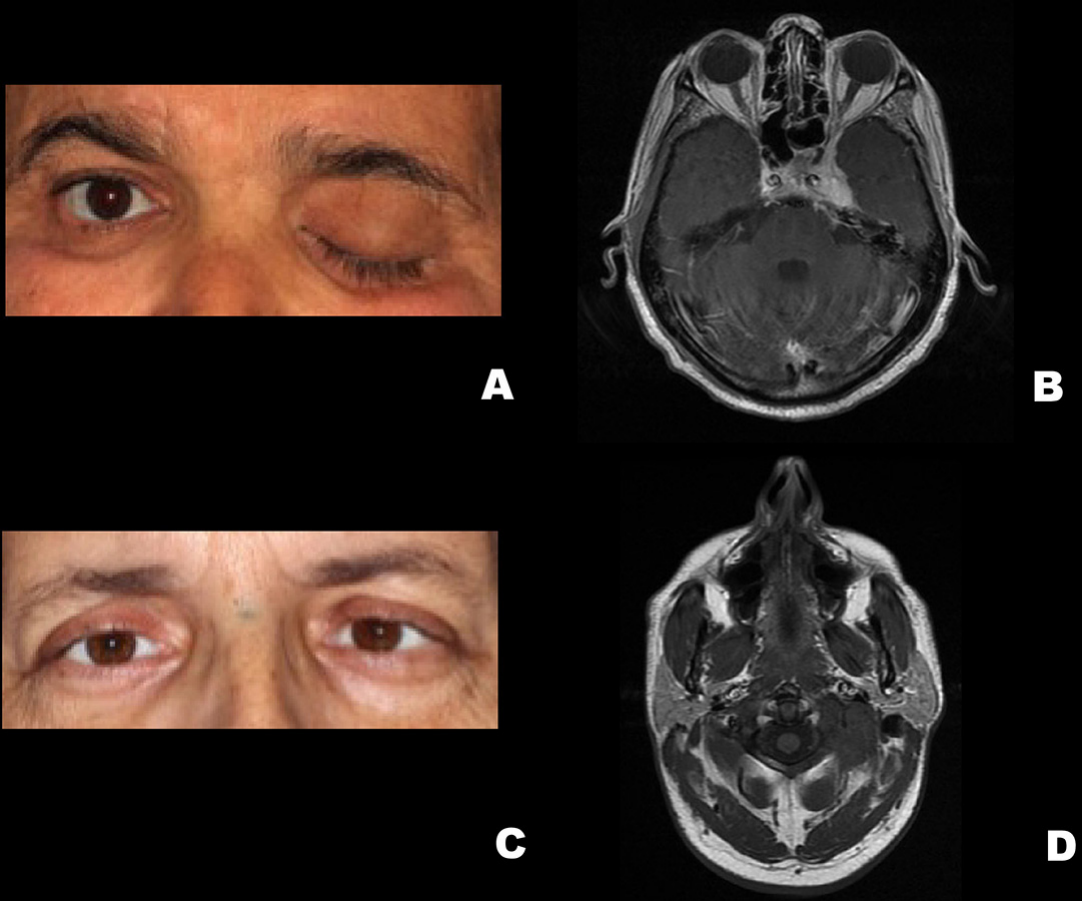
Patient 3. A. Extraoral clinical aspect showing left eyelid ptosis. B. Axial IMR (T2-weighted) demonstrating hyperintense image at the skull base causing enlargement of the left optical canal. Patient 4. C. Extraoral clinical aspect revealing fixed globe in the left eye. D. Axial IMR (T2-weighted) demonstrating a mass involving the left sphenoid bone and causing hypoglossal canal obliteration.

Four patients with a previous history of head and neck carcinomas were included in this case series. All of the patients were men, with a mean age of 61.2 years at the time of diagnosis, presenting advanced stage tumors with a previous history of tobacco and alcohol consumption. Detailed demographic and clinicopathological information are presented in  Table 1 and  Table 2. Illustrative images of the patients are presented in figures 1,2 and 3.

**Table 1:**
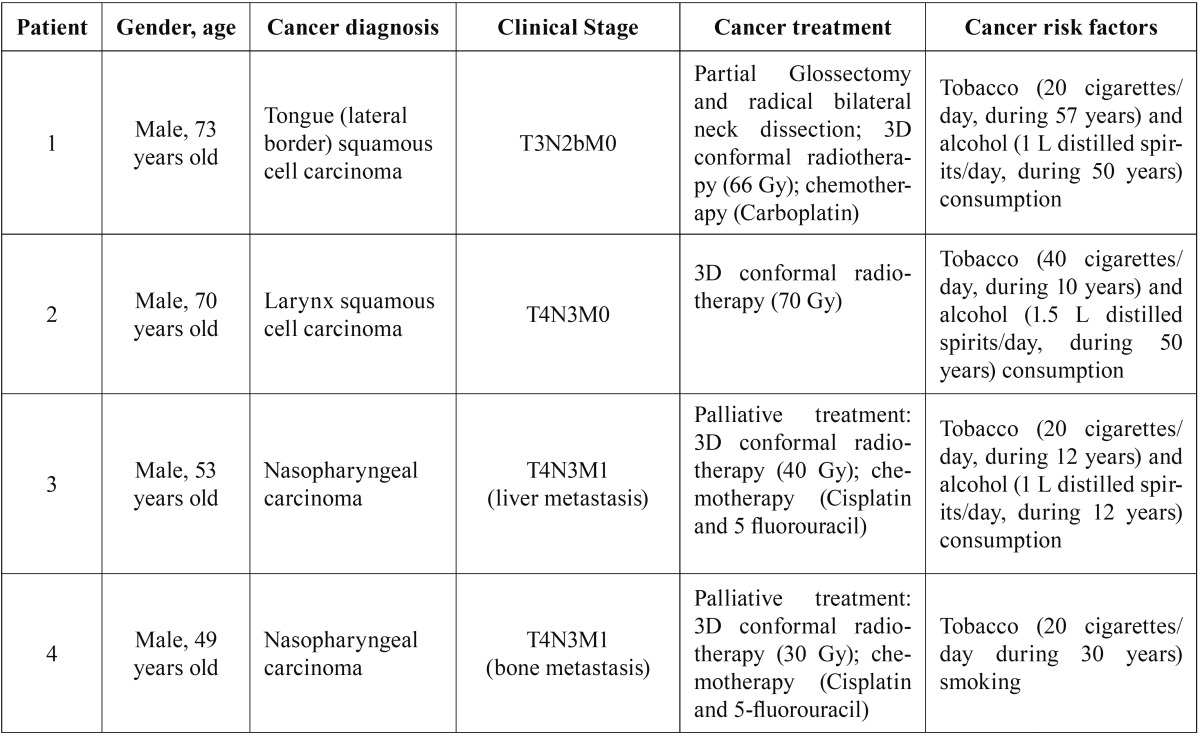
Demographic and clinicopathological features of head and neck cancer patients diagnosed with orbital apex syndrome.

**Table 2:**
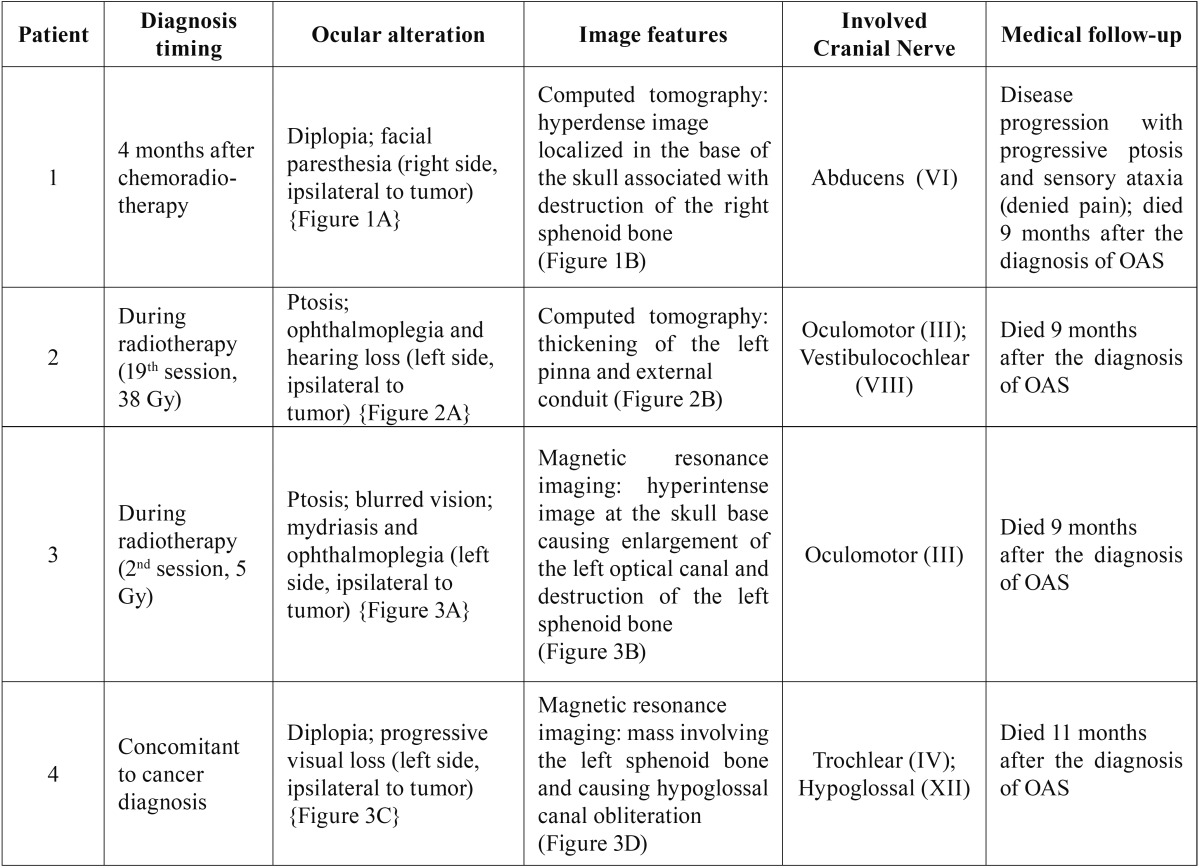
Clinicopathological features of orbital apex syndrome in head and neck cancer patients.

## Discussion

HNC is the sixth most common cancer worldwide (6,7), accounting for an annual incidence of approximately 700,000 cases (nearly 5% of all cancers) and almost 375,000 deaths (7). HNC primarily affects men between the sixth and the seventh decades of life. Smoking and alcohol use are the major risk factors (8). In accordance with the above-mentioned literature, all the patients in the current series were elderly men with a previous history of tobacco and alcohol abuse.

HNC patients tend to present a loco-regional spread of disease; the most common site of metastasis is the regional neck lymph nodes. Distant metastasis from HNC is considered a rare event (9-11). Kowalski *et al.* (10) evaluated 2,327 patients diagnosed with HNC and found that only 3.8% of the patients had distant metastasis, while Alvi *et al.*, (9) reported a distant metastasis rate of 23%. Lung and bone were the most common distant sites for distant metastasis in both studies. Accordingly, advanced HNCs affected all of the patients included in the present case series.

The association between OAS and HNC is unusual. In this context, Whirth *et al.*, (5) recently reported a case of oral squamous cell carcinoma associated with ophthalmoplegia and severe general facial pain, leading to the diagnosis of OAS. Aryasit *et al.* (2) performed a retrospective review of patients diagnosed with OAS and observed that 48% of which had associated neoplasias were lymphomas (37.5%) was the most common tumor followed by meningiomas (29.2%). Apparently, the incidence of the association between HNC and OAS is currently unknown and, when judging the cases presented herein, one could suggest that this association might be unrecognized and might not be as rare as once believed.

Patients diagnosed with OAS usually present with visual impairment and ophthalmoplegia, including proptosis, ptosis and periorbital pain. The diagnosis of OAS is clinical; however, a detailed history and physical examination should be performed (12,13). Imaging is important in discerning the causative etiology (2,3). In the current case series, all the patients presented ocular altera-tions, including two patients presenting diplopia and two other presenting ptoses.

Most of the patients diagnosed with distant metastasis of HNC are considered to be incurable and are often treated by palliative care protocols, such as chemotherapy or radiotherapy (10). According to Alvi *et al.* (9), the average overall survival rate after the diagnosis of distant metastasis of HNC is 5 months, while Kowalski *et al.* (10) reported a rate of 1.8 months in patients with bone metastasis. In the present case series, the average overall survival rate after the diagnosis of skull metastasis (or infiltration) associated with OAS was 9.5 months. The present results reinforce previous evidence that OAS is a marker of poor disease outcome in cancer patients and emphasize the need for prompt identification of clinical signals of OAS in HNC patients (2).

Overall, up to 80% of all HNC patients are diagnosed with advanced stages of the disease, presenting poor prognoses and requir-ing aggressive multimodality treatment (7,12,14) in association with multidisciplinary supportive care where dental oncologists may contribute to the early diagnosis of the disease relapse or relevant complications of tumor progression, such as OAS. Thus, this case series highlights the importance of interactions among dentists, oncologists, and physicians involved in HNC treatment to improve the early recognition of OAS and promote patient prognosis.
